# Impressions of a Novice

**Published:** 1912-12-28

**Authors:** 


### IMPRESSIONS OF A NOVICE.

Goodness knows how they came by it, but they enjoy with all its privileges that state of the chaste mystic who seeks no Heaven and fears no Hell—chiefly the latter. There is little of chaste mysticism about them, however. They are a well-set-up, formidable lot, and they enjoy life at any cost. If they retain their *joie de vie* it will be a tonic for their patients' nerves and spirits more valuable than a Sheffield full of cutlery or German Ocean—I mean Bosphorus—full of medicine. And when one comes to think of it they are singularly the right people in the right place; for they are almost a caste on their own with this characteristic—that ill-health in mind, body, or estate does not have the effect, as it often does with others, of making them unsociable and separate.

My first experience was a biology lecture. One burly Briton with a huge chest thought, as he was an old hand, that he ought to put us at our ease. We were listening in the most solemn way when he interrupted the lecturer with a request for information on some remote point little connected with the subject. Having received a careful and learned answer he replied : "But still, that's neither here nor there, is it, Sir?" That lecturer used to let us do as we liked, but he managed to get a high percentage of passes. He had the soul of a poet and invested biology with a charm all his own. But he has left us now; I often wonder if we really did worry him or not; I suspect it was the dog-fish. We *would* throw them about, and one hit him one day. We all mourned him.

It's a fearsome thing—*a viva.* They keep you waiting a long time on a draughty staircase—you get colder and colder and shiver more and more, and stand staring at the door where someone has written : "Abandon hope all ye who enter here." And, when you get in, the reaction of the warm room makes you shiver more. One angry examiner sits and makes faces while the other asks ridiculous questions. It isn't right. They start at an hour when ordinary people should still be in bed, and continue to a like one. No wonder the examiners get cross. In spite of this, however, it is obvious that they are trying to help one to do as well as possible. Indeed, these examinations are the reverse of what most examinations used to be some years ago, when not only the requisite knowledge was tested but the student's ability to avoid pitfalls and to resist efforts to disconcert him. The excellent relations now existing are aptly shown by the following incident. It was at a written examination in the afternoon, and tea was brought in for the presiding examiner, a venerable gentleman of most friendly aspect. Spontaneously throughout the hall there was a tapping of heels on the floor. It stopped without the examiner having to remonstrate.

If there is a lot of kindness in a hospital there is a lot of friendliness in a medical school. The "atmosphere" is altogether excellent. The lecturers seem to be there because they like it. The students are all keenly interested. There is something finely impersonal about it all—an air of absorption in the place and its work to the pleasant obliteration of all other attractions. The novice feels he has found something that suffices; something in which he can live and gratify his aspirations, and which will exercise his best energies.

The doctors' calling makes them great. They have found one of Nature's secrets—that a new phase of consciousness accompanies disease—but they have a silent Freemasonry. If death is the great leveller disease is the great chastener, and as all the world loves a lover, so all doctors love a sick man. It is not the fees—it is patent to the meanest physiognomist that they make money to doctor rather than doctor to make money. Quite apart from the promptings of natural humaneness, is there not a strong and perfectly obvious and distinct fascination which emanates impersonally from the patient at the centre and pervades the whole medical sphere? Can it be a form of that universal mystic charm which is always associated with the unknown life after death? And perhaps the doctors' reputation for materialism is due to this also; for if they have found so excellent a "way" they will refuse all others, of which the adherents forthwith label them materialists, atheists, and so on. It may be objected that as no one ever discusses it it does not exist—a frequent form of criticism this. But it is a fact that the finer the type of mysticism the less do its bearers care to speak of it. Pearls are not to be cast before the uninitiate. Many diseases are well understood and have a regular course and treatment, subject to familiar variations, with corresponding recognised modifications of treatment. These should be uninteresting if interest lay in the physical phenomena only; but they are not so. There is undoubtedly a psychological interest too. How few doctors are not aware of it deep down in their minds and have not the secret ambition that medical science will one day reach the specific for death itself! And why not? Material for the heavenly life is generated in earthly life—often obviously it is an advantage permanently to maintain a physical body and earthly life in which to generate more and better material for heavenly life. There is no need either for the destruction of the physical body for heaven to be enjoyed. A great physician, I think it was Sir Walter Foster, said in a public speech that he looked forward to a time in the near future when disease would be entirely banished by medical science. That is the first step. But death is a mental and moral process, as all but dullards know. Is it not a disease which develops progressively throughout life from the adult stage? Some of its symptoms are easy. Observe an old man—he is busy grouping isolated acts, or rather, the memory of them. He contemplates the relation of each to the others. This process is directly antagonistic to the nature of the physical body, which is singleness; witness the child. Civilisation is a fight against death in this sense, too, and tends to subdivide the work of its maintenance so that each individual has a single kind of work, and if he puts his heart into it he is relieved from further responsibility. The physical life of the savage is like the mental life of an aged person, for he is his own butcher, baker, builder, soldier, etc. True, the civilised man has many other actions, but unlike his staple industry they are, in point of necessity, negative like those of a child, and would be performed, like those of a healthy unfettered child, with unalloyed unreasoning joy if civilisation were complete. Such acts, then, would be fortifying physical life—a specific for the state which brings death to the aged, however healthy, at the present time.

Disease, unlike health or old age, derives a psychological interest from the fact that there is a temporary mobilising and opposition of the forces of life and death. The two are then easier to study; for in the absence of illness death is working latently and life is about its business.

Notoriously scientists are dissatisfied with orthodox religion. All science is addressed to human weal and the logical end of it is permanent physical life. The idea of permanence is the satisfying element in every religion. May not scientists be deriving their religious satisfaction from this idea? For all men face the religious question and death, and scientists are not exempt. Then may not the doctor's work be his religion too—and an unselfish one at that? Are we always to be impotent? Why! Faith will move mountains; but this is more—the devotion of science, and that religious. Will it not suffice?

